# Decoloniality in the Psychedelic Research Space

**DOI:** 10.1192/bjo.2022.237

**Published:** 2022-06-20

**Authors:** Juliet Raphael

**Affiliations:** Queen Mary University of London, London, United Kingdom. University of East Anglia, Norwich, United Kingdom

## Abstract

**Aims:**

Since the 1950's, there has been increasing interest in the potential of the psychedelic experience to generate an enhanced state of emotional well-being in those suffering from a range of mental disorders. Following the so-called ‘War on Drugs’, much of this research was curtailed until a new surge of interest resulting in a ‘psychedelic renaissance’. This has come at a time where powerful institutions, including the medical sphere, are being asked to address their oppressive and damaging pasts; these narratives bear particular relevance to psychedelic research given the widespread use of entheogenic plants as medicines and tools for spiritual healing amongst indigenous groups worldwide, and the political history of the War on Drugs. The aim of this study was to explore how those in the psychedelic community have come to understand what it means to ‘decolonise’ this space and to situate these conversations within existing literature.

**Methods:**

Semi-structured interviews were conducted with 10 participants who were recruited using theoretical and snowball sampling. Data collection and analysis were carried out from a critical theoretical standpoint, further borrowing from aspects of constructivist grounded theory methodologies. This involved open coding of existing literature to devise an interview guide, followed by coding of interview transcripts to generate several key themes as they emerged from the primary data.

**Results:**

Analysis of the data generated 8 sub-themes, which were then combined to create the 4 main themes;

- The Making of a ‘New’ Medicine
- Scientism and Spirituality
- Appreciation vs Appropriation
- Beyond Decriminalisation

A theoretical framework which sought to bridge decolonial and social justice approaches was used to understand how participants made links between these two related but distinct concepts. Foucauldian theories of biopower were also explored and integrated into the discussion. Participants assigned wide-ranging meaning to the concept of decoloniality in reference to psychedelic research, though there were calls not to appropriate the term itself and senselessly apply it to any issues of injustice.

**Conclusion:**

The study demonstrated the participants’ willingness to engage in a discussion which explored some uncomfortable truths regarding psychedelic research. There was a suggestion amongst some participants that the space can never be truly decolonised given the capitalist and neo-colonial manifestations within the current space. Future research should seek to facilitate more critical discussion of the epistemic, material, and geopolitical injustices which exist, and critical indigenous methodologies offer a meaningful way of understanding and undoing the hierarchical power structures currently at play.

